# Comprehensive Evaluation of the Oral Health Status, Salivary Gland Function, and Oxidative Stress in the Saliva of Patients with Subacute Phase of Stroke: A Case-Control Study

**DOI:** 10.3390/jcm9072252

**Published:** 2020-07-15

**Authors:** Piotr Gerreth, Mateusz Maciejczyk, Anna Zalewska, Karolina Gerreth, Katarzyna Hojan

**Affiliations:** 1 Private Dental Practice, 57 Kasztelanska Street, 60-316 Poznan, Poland; piotrger@hotmail.com; 2 Department of Hygiene, Epidemiology and Ergonomics, Medical University of Bialystok, 2C Adama Mickiewicza Street, 15-022 Bialystok, Poland; 3 Experimental Dentistry Laboratory, Medical University of Bialystok, 24A Marii Sklodowskiej-Curie Street, 15-276 Bialystok, Poland; azalewska426@gmail.com; 4 Department of Risk Group Dentistry, Chair of Pediatric Dentistry, Poznan University of Medical Sciences, 70 Bukowska Street, 60-812 Poznan, Poland; 5 Department of Rehabilitation, Greater Poland Cancer Centre, 15 Garbary Street, 61-866 Poznan, Poland; katarzyna.hojan@op.pl; 6 Department of Rehabilitation, Greater Poland Provincial Hospital, Juraszow Street, 60-479 Poznan, Poland

**Keywords:** cerebral stroke, salivary biomarkers, redox status, oxidative stress, antioxidants

## Abstract

This is the first study to assess, comprehensively, the oral health status; salivary glands’ function and enzymatic and non-enzymatic antioxidant defense; and oxidative damage to proteins and lipids in the non-stimulated (NWS) and stimulated (SWS) whole saliva of stroke patients. The study included 30 patients in the subacute phase of the stroke and an age and gender-matched control group. We showed that the activity of antioxidant enzymes (catalase and salivary peroxidase) was significantly higher in both NWS and SWS of stroke patients, similarly to uric acid concentration. However, in the study group, the reduced glutathione (GSH) concentration in SWS decreased. The contents of protein glycooxidation products (advanced glycation end products (AGE) and protein oxidation products (AOPP)) and lipid hydroperoxides were significantly higher in NWS and SWS of stroke patients. In the study group there was also a decrease in stimulated saliva secretion and total protein content. Interestingly, products of protein and lipid oxidation correlate negatively with SWS flow. The ROC analysis showed that salivary GSH with 100% specificity and 100% sensitivity differentiates the analyzed groups (AUC = 1.0). To sum up, in subacute stroke patients there are redox imbalances and oxidative damage to proteins and lipids in non-stimulated and stimulated saliva. Stroke patients also suffer from salivary gland dysfunction.

## 1. Introduction

Currently, there is a global epidemic of strokes [1]. As demonstrated in epidemiological studies, cerebral stroke is considered the second single most prevalent cause of death of individuals over the age of 60, the second most frequent cause of dementia, and the most common reason for permanent disability [2]. Since stroke is a serious worldwide health problem, there is an urgent need to develop new diagnostic and therapeutic methods. The phases of stroke are divided up into hyper-acute (from onset to first 24 h), acute (1–7 days), early subacute (7 days to 3 months), late subacute (3 to 6 months) and chronic stroke phase (over 6 months) [3,4]. The mechanisms thought to play a role during the subacute phase involve the resolution of the decrease in hypometabolism of structurally normal regions remote from the infarct due to disruption of a functional pathway to those regions [5].

During the last two decades, remarkable advances in oxidative stress research have been seen, especially regarding ischemic brain injury [6]. Oxidative stress (OS) is induced by the increased formation of reactive oxygen (ROS) and nitrogen species (RNS), which is not balanced by antioxidant systems of the body [7]. As a result, cell components (i.e., proteins, lipids, and DNA) are damaged [8]. In stroke patients, OS influences the integrity of the genome (resulting in DNA lesions and neuronal cell death) and impairs neurological recovery following the incident [6]. It was shown that the accumulation of protein oxidation products leads to morphological changes in the brain tissue and elevates the production of pro-inflammatory cytokines. Interestingly, advanced glycation end products (AGE) and advanced oxidation protein products (AOPP) increase the production of ROS by inducing NADPH oxidase (NOX) activity, which is the primary source of free radicals in neurons and glial cells [9]. However, oxidative stress can also affect salivary gland function. This is not surprising because the oral cavity is the only place in the body exposed to so many pro-oxidant factors, such as diet, xenobiotics, dental materials, and air pollutants [7,10]. It was shown that ROS/RNS cause oxidation of proteins and lipids of the salivary glands responsible for morphological and functional changes in their parenchyma. Indeed, oxidative stress-related dysfunction of salivary glands and changes in the qualitative composition of saliva have been demonstrated in many systemic diseases, including, in particular, neurological disorders [11]. However, salivary redox homeostasis has still not been assessed in stroke patients. Indeed, it has been demonstrated that acute ischemic stroke increases OS level and decreases antioxidant enzymes in serum/plasma samples [12,13]. However, despite the confirmed participation of OS in stroke at a central level, there is no information concerning the research on salivary oxidative stress. Moreover, little is known about the diagnostic utility of salivary redox biomarkers. The results of current studies show the high diagnostic value of salivary redox parameters in the diagnosis of such conditions as chronic kidney disease [14,15,16], chronic heart failure [17], hypertension [18], obesity [19,20], Alzheimer’s disease, and dementia [11,21,22]. Considering the increasing incidence of strokes, studies involving salivary redox homeostasis in these patients are necessary.

Previous studies indicate a link between oral health and general condition of stroke patients. It has been demonstrated that subjective oral health was significantly poorer in stroke cases (such as tooth loss and dental caries experience), according to ambulation level and functional independence [23]. Additionally, some studies have shown a relationship between periodontal disease and the occurrence of strokes [24]. Nevertheless, although the need for constant dental care in stroke patients is indicated, the causes of oral diseases/salivary hypofunction in this group are still unknown. Considering the key role of oxidative stress in the pathogenesis of oral and salivary gland diseases, the aim of the present study was to assess the oral health status, the salivary gland function, the enzymatic and non-enzymatic antioxidant defense, and the oxidative damage to proteins and lipids in the non-stimulated and stimulated saliva of patients after cerebral stroke.

## 2. Material and Methods

### 2.1. Ethical Issues

The study was approved by the Ethics Committee of the Poznan University of Medical Sciences (resolutions 59/19 and 890/19).

### 2.2. Study Participants

The research was carried out between June and September 2019, in Bonifraterskie Centrum Zdrowia (health center) in Piaski–Marysin (Piaski, Poland). The center hospitalizes individuals after cerebral stroke, from different provinces of Poland. All patients were informed about the purpose and procedures of the study. Full written consent was obtained from all participants in accordance with the Declaration of Helsinki. The participation of each individual in the study was voluntary. One experienced medical doctor, a neurorehabilitation specialist, qualified all the patients for the examination according to study criteria.

### 2.3. Study Criteria

The criteria to include stroke sufferers in the study were as follows: age of consent (>18 years); good general condition; confirmed cerebral infarction or cerebral hemorrhage based on CT and magnetic resonance imaging (MRI); recovery from acute phase of ischemic or hemorrhagic stroke in all brain areas; first admission to cure stroke unit was more than 5–6 (to 10) h from the onset of the first neurological symptoms; consciousness and giving of written and informed consent for oral examination and sampling of saliva; adequate capacity to follow instructions, i.e., ability to collect saliva sample, understanding how to perform the procedure, and being able to answer questions during the examination.

The criteria to exclude subjects from the research were: patients under the age of 18, unconfirmed cerebral infarction or cerebral hemorrhage with CT and magnetic resonance imaging (MRI), ischemic stroke treated with thrombolysis or thrombectomy, stroke recurrence during subacute phase, unconsciousness and inability to give informed consent for oral check-up and saliva sampling, legal guardianship, incapability to collect saliva sample, insufficient cooperation due to language/cognitive deficits, heart failure resting oxygen saturation (SaO_2_) ≤ 92%, autoimmune disease (systemic lupus erythematosus, rheumatoid arthritis), cardiovascular disease (angina or uncontrolled hypertension) or lung disease (chronic obstructive pulmonary disease), women suffering from malnutrition (body mass index <18 kg/m^2^) or weight loss of >10% during the previous 3 months, patients with psychiatric or cognitive disorders.

Additionally, smokers and patients taking vitamins and dietary supplements for the last 3 months were excluded from the research.

### 2.4. Study Group

**Figure 1:**
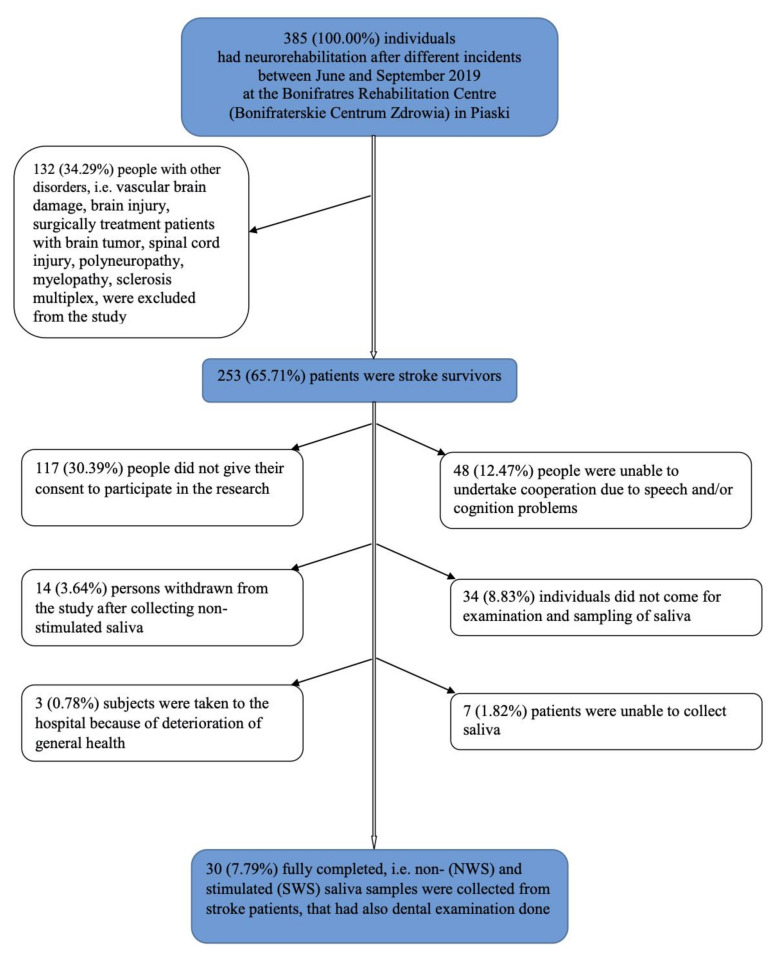
Study population flow chart. NWS: non-stimulated whole saliva; SWS: stimulated whole saliva.

The study group consisted of stroke patients in subacute phase who were selected from patients in the Bonifraterskie Centrum Zdrowia. Out of 385 individuals that were subjects in the neurorehabilitation ward following different incidents, including vascular brain damage, brain injury, surgically treated patients with brain tumor, spinal cord injury, polyneuropathy, myelopathy, and sclerosis multiplex, 253 (65.71%) patients were stroke survivors. As many as 48 (12.47%) people were unable to cooperate, i.e., to give conscious written informed consent and/or communicate. Moreover, 117 (30.39%) people did not give their consent to participate in the research, 34 (8.83%) individuals did not appear for examination and sampling of saliva, even though they gave their consent and were reminded 3–4 times, 14 (3.64%) persons withdrew from the study after non-stimulated saliva was collected because of psychological and/or physiological tiredness, 7 (1.82%) patients were unable to collect saliva because of general problems in understanding the procedure due to language and cognitive deficits, and 3 (0.78%) subjects were taken to the hospital because of deterioration of general health. Finally, 30 (7.79% of all individuals that were rehabilitated at the hospital; 11.86% of stroke survivors) fully completed examination (Figure 1).

Study group patients were admitted to the neurorehabilitation unit in a subacute phase of stroke, directly from the hospital, immediately after the acute phase cessation. They were evaluated by a medical doctor, and then they were subjected to comprehensive individual and similar rehabilitation. Most patients were able to cooperate and communicate and they understood instructions. Most participants followed the same diet divided into a baseline diet for most of the patients or a diet for diabetes mellitus individuals. All the meals were prepared in this hospital and were distributed at the same time daily.

Data on an individual’s general health status and condition were taken from patient’s files, and included: gender, age, medical history, time since diagnosis of cerebral stroke, and medication used.

Additionally, to measure functional status we used the following scales:

1. Addenbrooke’s Cognitive Examination III (ACE III) was used to differentiate patients with and without cognitive impairment [25].
2. The functional independence measure (FIM) was used to explore individual’s physical, psychological and social functioning [26].
3. The Barthel Index (BI) was used to measure performance in activities of daily living (ADL) [27].
4. The Berg Balance Scale (BBS) was used to determine patient’s ability (or inability) to safely balance during a series of predetermined tasks [28].

### 2.5. Control Group

The control group consisted of 30 healthy people who were similar to the study population in terms of age and gender, status of dentition, oral hygiene, and periodontium, and consisted of individuals reporting for dental examination to the Department of Restorative Dentistry of the Medical University of Bialystok (Bialystok, Poland) from March 2017 to September 2017. Clinicians provided medical clearance prior to control group involvement in the study. Patients in control group were given standard physical activity recommendations and followed a normal, balanced diet (not restricted).

### 2.6. Oral Examination

The participation of each individual in the study was voluntary and the examination was performed in a separated room, directly after saliva collection. Dental examination was carried out, in artificial lighting, according to the World Health Organization criteria [29]. The dentition was assessed with the use of a plane mouth mirror and a dental probe, while the patient was seated in a chair with their head resting against the wall, and the examiner stood in front of the chair. Each tooth was assessed and scored as sound, decayed (DT), extracted due to caries (MT), or filled because of carious process (FT). The data obtained were used to calculate the DMFT index, which expresses dental caries experience, and it is the sum of DT, MT, and FT. Dental caries prevalence was calculated as a percentage of subjects with DMFT > 0. Gingival index (GI) [30] and plaque index (PlI) [30] were also determined.

The dentition was evaluated by two dentists (P.G. and K.G.), after non-stimulated (NWS) and stimulated (SWS) whole saliva collection, and after previous training and calibration by experienced dental specialist (A.Z.). The intra-examiner and inter-examiner agreement for DMFT was assessed by another dental check-up in 10 patients after two weeks, with a κ that amounted to 1.00 and 0.96, respectively; whereas for GI and PlI κ was 0.96 and 0.92 and 0.92 and 0.96, respectively.

### 2.7. Saliva Sampling

The studied material was total mixed non-stimulated saliva (NWS) and stimulated saliva (SWS), and both types of samples were collected via spitting. This oral bioliquid was collected between 7:30 a.m. and 9:00 a.m. from individuals who had not practiced intensive physical activity for the preceding 12 h. Patients were instructed not to intake any solid and liquid food, other than clean water, at least 2 h before saliva sampling. They were also indicated not to carry out any oral hygiene procedures (i.e., teeth brushing, mouth rinsing, gum chewing, etc.). Since all subjects were in subacute phase after stroke incidence, they had to take medications within 8 h prior to sampling, however, the time from last dose of any drug was minimally 2 h. The subjects from the control group had not taken any drugs at least 8 h before the saliva collection [22]. The oral cavity was rinsed two times with distilled water at room temperature before saliva was collected, in order to avoid possible contamination from other sources. The oral bioliquid was collected in a separate, private room, after at least a 5-min adaptation to the environment. During the research, the patients were seated in an adjustable chair, individually adapted to the height of each person, with the head slightly bent downwards, and resting in comfortable position. The individuals tried to limit the movements of their lips and face. The samples of saliva that were collected during the first minute were ejected. Saliva was accumulated into a sterile Falcon tube that was placed in a container with ice (temperature of approximately −80 °C). The secretion of saliva was stimulated by application of 10 μL of 2% citric acid on the central part of the tongue every 30 seconds. The non-stimulated saliva was collected for 10 min to avoid psychological and/or physiological tiredness of the patients, whereas SWS was sampled in the same manner for 5 min. Directly after sampling of saliva, the volume of liquid was measured using a calibrated pipette with the accuracy of 0.1 mL [31]. The minute flow of SWS and NWS was assessed by dividing the volume of saliva by the time necessary for its secretion, and expressed in mL/min. Instantly after saliva was collected, it was centrifuged (+4 °C, 20 min, 3000 × g; MPW 351, MPW Med. Instruments, Warsaw, Poland). Butylated hydroxytoluene (BHT, Sigma-Aldrich, Saint Louis, MO, USA) was inserted to the obtained supernatants, in the amount of 10 μL 0.5 M BHT in acetonitrile (ACN)/1 mL of saliva, to protect the samples from oxidation processes [32]. The samples of saliva were frozen at −80 °C and stored for no more than three months for further research. 

The saliva sampling was performed in the rehabilitation center during summer time, i.e., between June and September, to maintain similar weather condition outside.

### 2.8. Redox Assays

#### 2.8.1. Salivary Antioxidants

Both antioxidant enzymes and non-enzymatic antioxidants were analyzed to evaluate the antioxidative barrier of saliva. The activity of salivary peroxidase (Px, EC 1.11.1.7) was assessed colorimetrically at 412 nm in a reaction with Ellman’s reagent (5,5’-dithiobis-2-nitrobenzoic acid, DTNB) [33]. The activity of salivary catalase (CAT, EC 1.11.1.6) was assessed colorimetrically at 240 nm by measuring hydrogen peroxide decomposition [34]. One unit of CAT activity was defined as the quantity of the enzyme catalyzing decomposition of 1 mM of hydrogen peroxide per 1 min. The activity of salivary superoxide dismutase (SOD, E.C. 1.15.1.1) was assessed colorimetrically at 480 nm by measuring the inhibition rate of adrenaline oxidation [35]. One unit of SOD activity was defined as the quantity of enzyme inhibiting adrenaline oxidation by 50%.

The concentration of salivary reduced glutathione (GSH) was assessed colorimetrically at 412 nm using the enzymatic reaction with DTNB [36]. The concentration of salivary uric acid (UA) was assessed colorimetrically at 630 nm using the commercial kit (QuantiChromTM Uric Acid DIUA-250; BioAssay Systems, Harward, CA, USA), according to the manufacturer’s instructions.

The precisions of these measurements, expressed as coefficients of variation (CV), were <3.5%, 4%, 3%, and 4%, respectively.

#### 2.8.2. Salivary Redox Status

The level of total antioxidant capacity (TAC) of saliva was assessed colorimetrically at 660 nm using 2,2-azinobis-3-ethylbenzothiazoline-6-sulfonic acid (ABTS) radical cation and 6-hydroxy-2,5,7,8-tetramethylchroman-2-carboxylic acid (Trolox) as a standard [37]. The level of total oxidant status (TOS) was assessed bichromatically (560/800 nm) based on the oxidation of Fe^2^^+^ to Fe^3^^+^ in the presence of the oxidants contained in the saliva [38]. The precisions of TAC and TOS assay (CV) were <4% and <2%, respectively. Oxidative stress index (OSI) was calculated as TOS to TAC ratio: OSI = TOS/TAC × 100 [39].

#### 2.8.3. Salivary Oxidative Stress

Oxidative damage to salivary proteins (AGE, AOPP) and lipids (LOOH) was evaluated. The content of advanced glycation end products (AGE) was assessed fluorimetrically at 350/440 nm by measuring AGE-specific fluorescence [40]. Immediately before the assay, saliva samples were diluted (1:5, v:v) in 0.02 M phosphate-buffered saline (PBS), pH 7.4 [41]. The concentration of advanced oxidation protein products (AOPP) was assessed colorimetrically at 340 nm by measuring the iodide ion oxidizing capacity of the saliva [40]. Immediately before the assay, saliva was diluted (1:5, v:v) in 0.02 M PBS [41]. The concentration of total hydro-peroxides (LOOH) in saliva was assessed colorimetrically at 560 nm based on the reaction of xylenol orange with Fe^3^^+^ (resulting from Fe^2^^+^ after its oxidation by LOOH) [42].

The precisions of these measurements (CV) were <4%, 3%, and 5%, respectively.

### 2.9. Statistical Analysis

Statistical analysis was carried out using GraphPad Prism 8.3.0 for MacOS (GraphPad Software, La Jolla, CA, USA) and R software 4.0.2 for Windows. The Shapiro–Wilk test was used to check the normal distribution of results, while the Leven test to check the homogeneity of variance. The results were expressed as means ± SDs. A paired *t*-test was used to compare 2 groups. The ANOVA variation analysis with Tukey’s post hoc test was used to compare 3 groups. Multiplicity adjusted p value was also calculated. The Chi-square test was used for comparisons involving categorical variables. The assumed level of statistical significance was *p* < 0.05.

Correlation between the results was assessed using the Pearson correlation coefficient. Additionally, multivariate analysis of the simultaneous impacts of many independent variables on one quantitative dependent variable was made by the means of linear regression. Stroke, gender, age, ACE III, BI, FIM, BBS, NWS flow, and SWS flow were included as independent variables; 95% confidence intervals (CI) were reported along with regression parameters. To assess the diagnostic utility of salivary redox biomarkers, the area under the curve (AUC) and confidence intervals were determined based on receiver operating characteristic (ROC).

The number of patients was determined a priori based on the previous pilot study (*n* = 15). For this purpose, online sample size calculator (ClinCalc) was used. The level of significance was set at 0.05 and power of study was 0.9. Variables used for sample size calculation were salivary flow rate (both NWS and SWS), activity/levels of some salivary antioxidants (Px, CAT, and UA), and concentration of some oxidative stress products (AOPP and LOOH). The minimum number of patients was 24 (for one group).

## 3. Results

### 3.1. Characteristics of Study and Control Groups

Thirty patients who suffered stroke in subacute phase were recruited for this research, aged between 34 and 84. Oral examination and saliva sampling were carried out between 45 and 50 days after incident of stroke (on average 46.78 days with SD = 4.71). Table 1 shows the descriptive and comparative findings referring to the demographic variables and habits of the patients according to the group. In six individuals, hemorrhagic type of cerebral stroke was diagnosed, while 23 had ischemic type, and one patient suffered from ischemic type that then secondarily transformed into hemorrhagic type. For most patients (86.67%) it was their first incident of the disorder, whereas for four (13.33%) individuals it was their second. Almost all cerebral stroke patients had other general disorders.

### 3.2. Oral Health Status

Patients after cerebral stroke showed high caries prevalence (100.00%); the mean DMFT index in this population amounted to 23.13 ± 7.32 (range 8–32); and the percentage of individuals with DMFT over 25 was 50.00%. Moreover, six (20.00%) patients were edentulous (Table 2).

None of the stroke sufferers had DMFT = 0, but 12 individuals did not have active caries (DT = 0) (data not shown in the table). The DMFT index was mainly dependent on the number of missing teeth, and then to a lesser extent on those with fillings and with caries. In total, it could be assumed that the changes approximately were situated on three surfaces of the tooth since mean DMFT was 23.13 ± 7.32, whereas mean DMFS amounted to 96.89 ± 44.09. Eight patients had healthy gingiva with GI = 0, and in half of them (four subjects) the occurrence of dental plaque was not observed (PlI = 0) (data not shown in the table). The percentage of patients with any dental prosthetic appliance was high and amounted to 93.33%; however, 50.00% of individuals still needed some prosthetic treatment.

### 3.3. Salivary Gland Function

Non-stimulated saliva secretion did not differ significantly between the groups of stroke survivors and healthy controls. The secretion of stimulated saliva was significantly decreased in the patients after stroke in comparison to the control group (Table 3).

Total protein concentration in non-stimulated saliva did not differ significantly between study and control groups. Total protein concentration in stimulated saliva of the stroke patients was significantly lower than in the controls (Table 3).

### 3.4. Enzymatic Antioxidants

**Figure 2:**
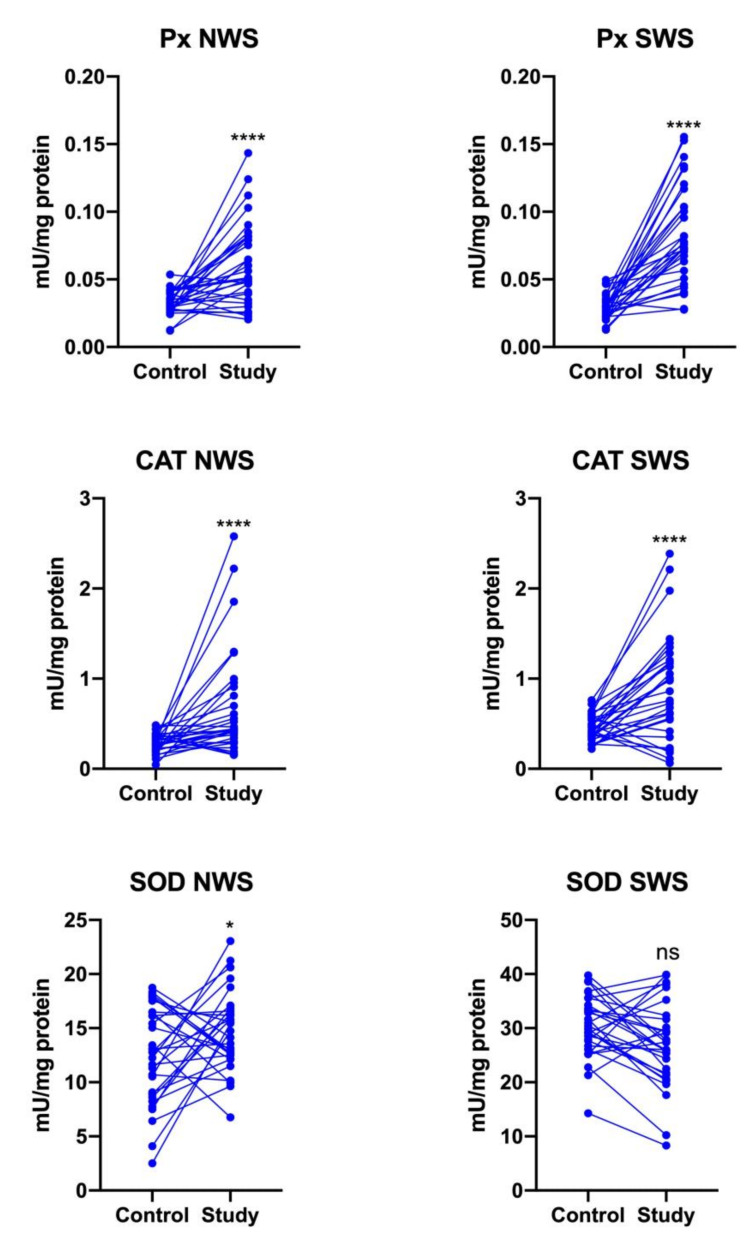
Enzymatic antioxidants in non-stimulated (NWS) and stimulated (SWS) whole saliva of stroke survivors and controls. Px: salivary peroxidase; CAT: catalase; SOD: superoxide dismutase-1; *—*p* < 0.05; ****—*p* < 0.0001; ns: non-significant.

Px (*p* < 0.0001) and CAT activity (*p* = 0.0004, *p* < 0.0001, respectively) were significantly enhanced in both the NWS and SWS of stroke patients compared to controls. SOD activity was significantly higher only in non-stimulated saliva of patients from the study group (*p* = 0.0473) (Figure 2).

### 3.5. Non-Enzymatic Antioxidants

**Figure 3:**
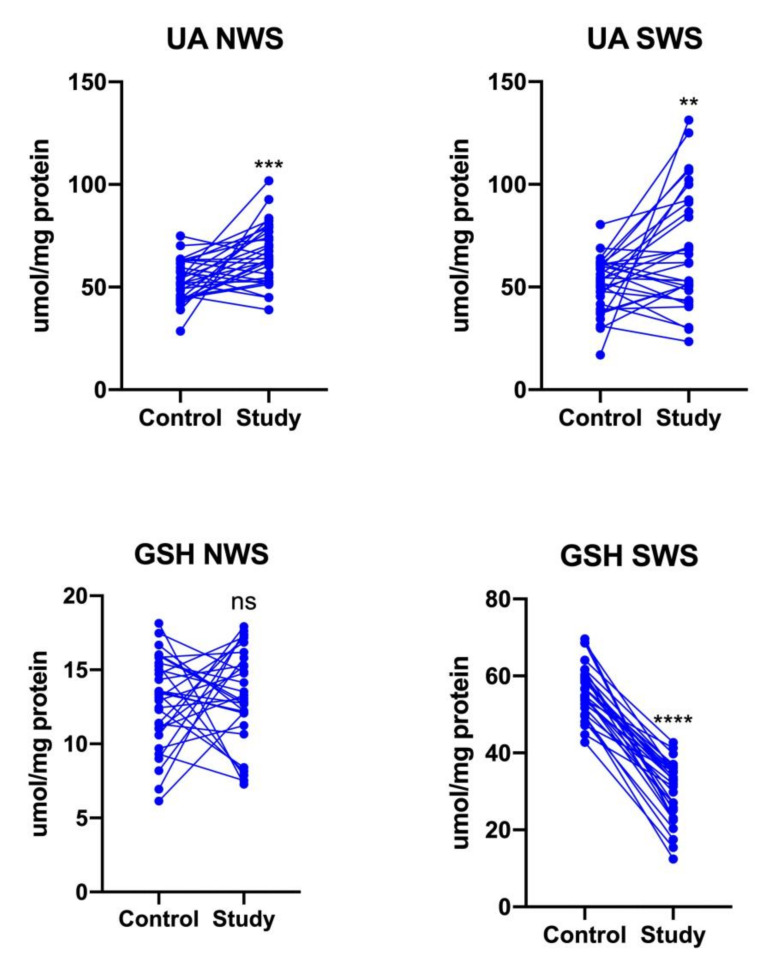
Non-enzymatic antioxidants in NWS and SWS of stroke survivors and controls. UA: uric acid; GSH: reduced glutathione; NWS: non-stimulated whole saliva; SWS: stimulated whole saliva; ns: non-significant; **—*p* < 0.01; ***—*p* < 0.001; ****—*p* < 0.0001.

The concentration of UA was significantly higher in both NWS (*p* = 0.0002) and SWS (*p* = 0.0062) of stroke patients compared to controls. On the other hand, GSH content decreased significantly only in stimulated saliva of patients from the study group (*p* < 0.0001) (Figure 3).

### 3.6. Redox Status

**Figure 4:**
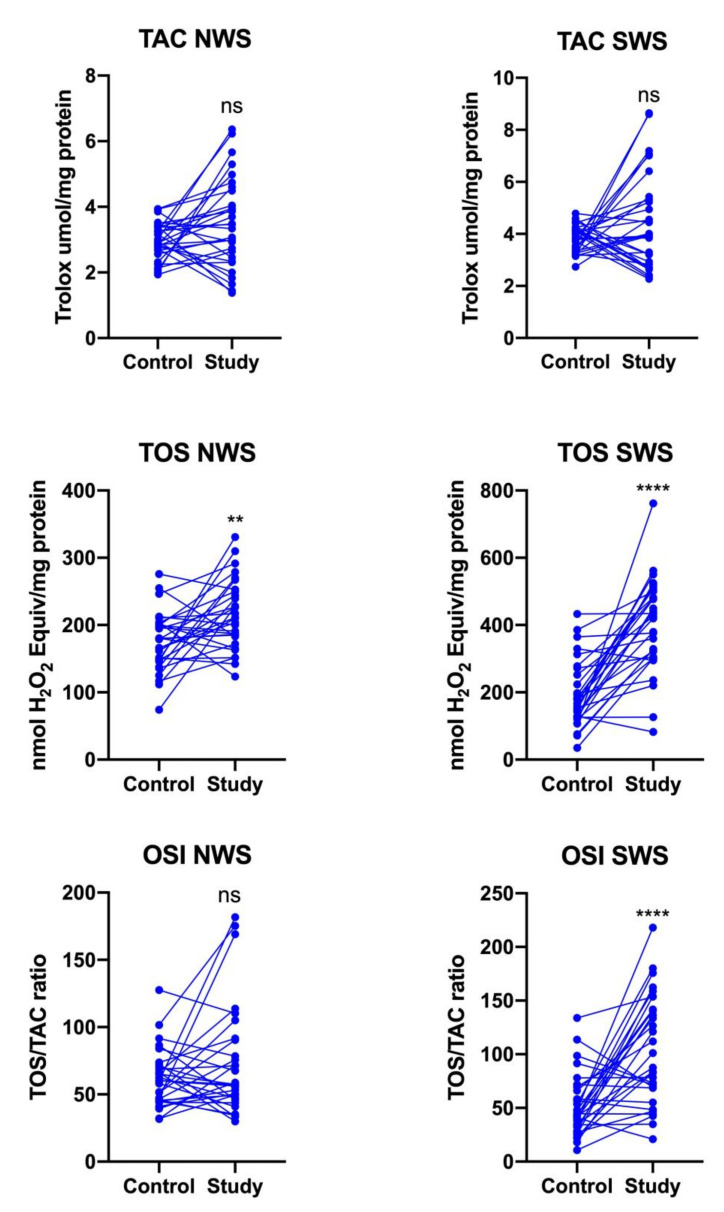
Redox status in NWS and SWS of stroke survivors and controls. TAC: total antioxidant capacity; TOS: total oxidant status; OSI: oxidative stress index; NWS: non-stimulated whole saliva; SWS: stimulated whole saliva; ns: non-significant; **—*p* < 0.01; ****—*p* < 0.0001.

The TAC level did not differ significantly between the study group and controls, while TOS was significantly higher in NWS (*p* = 0.0029) and SWS (*p* < 0.0001) of stroke patients. The oxidative stress index was significantly higher only in SWS of stroke patients compared to controls (*p* < 0.0001) (Figure 4).

### 3.7. Oxidative Stress

**Figure 5:**
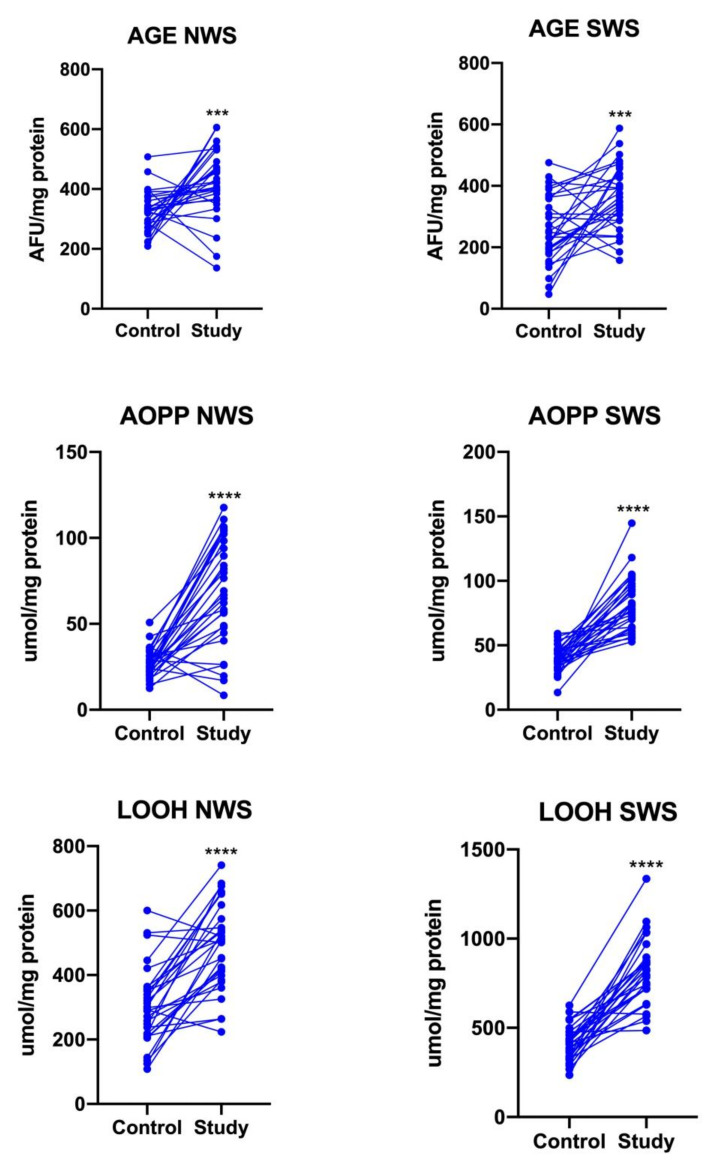
Oxidative damage in NWS and SWS of stroke survivors and controls. AGE: advanced glycation end products; AOPP: advanced oxidation protein products; LOOH: lipid hydroperoxides; NWS: non-stimulated whole saliva; SWS: stimulated whole saliva; ***—*p* < 0.001; ****—*p* < 0.0001.

The contents of protein (AGE, AOPP) and lipids oxidation products were significantly higher in both NWS and SWS of stroke patients compared to controls (*p* < 0.0001) (Figure 5).

### 3.8. Multifactorial Regression

The results of multifactorial regression analysis of salivary redox biomarkers in all patients are presented in Table 4. Interestingly, the variables affecting GSH concentration in SWS are the presence of stroke and the patient’s inability to safely balance during a series of predetermined tasks in BBS. However, the concentration of GSH does not depend on gender, age, or salivary secretion. The concentration of protein (AGE, AOPP) and lipid (LOOH) oxidation products depends on the salivary flow rate (in both NWS and SWS).

### 3.9. Correlations

Correlations between clinical parameters, redox biomarkers, and salivary flow rate are shown in Table 5. Interestingly, a positive relationship between glutathione concentration in SWS and cognitive functions in ACE III scale was shown. Salivary GSH also correlated positively with dynamic balance abilities in BBS. Moreover, the concentration of protein (AGE, AOPP) and lipid (LOOH) oxidation products in SWS correlated negatively with salivary flow rate (Table 5).

### 3.10. ROC Analysis

**Figure 6:**
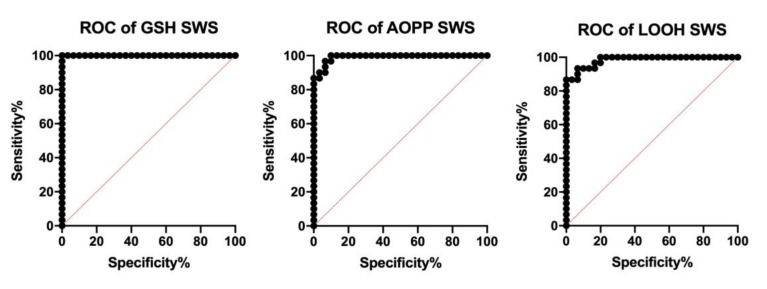
ROC curves of salivary GSH, AOPP, and LOOH in stimulated saliva of stroke patients compared to the controls.

In order to assess the diagnostic suitability, we also checked the sensitivity and specificity with which redox biomarkers differentiate stroke patients from controls. The results of the ROC analysis are presented in Table 6 and Table 7. Of all evaluated parameters, special attention was paid to the assessment of GSH concentration in SWS, which with sensitivity and specificity equal to 100% differentiates the study group from controls (AUC = 1.0). Salivary AOPP and LOOH are also characterized by high diagnostic usefulness (Figure 6).

### 3.11. Supplementary Material

Comparisons between the controls and hemorrhagic and ischemic stroke sufferers are presented in the Supplementary Material. The only parameter differentiating the types of stroke patients was SOD activity in NWS (Tables S1 and S2).

## 4. Discussion

The results of recent studies indicate the usefulness of salivary biomarkers in stroke diagnostics. Indeed, clinical relevance has been demonstrated for salivary cortisol [43,44], enolase [45], substance P (SP) [46], Il-1β (interleukin 1 beta), and MMP-8 (matrix metalloproteinase-8) [47]. However, there are no studies assessing salivary redox homeostasis. This research is the first to demonstrate disturbances of the antioxidant barrier and enhanced oxidative damage in the non-stimulated and stimulated saliva of stroke patients in the subacute phase of stroke. Some of the redox parameters (especially salivary glutathione) may be potential biomarkers in the non-invasive diagnostics of stroke. The subacute phase of stroke is also associated with salivary gland dysfunction.

Recent studies confirm the important role of oxidative stress in stroke pathomechanism [48,49,50]. The risk factors for cerebrovascular diseases are aging, hypertension, obesity, diabetes, alcohol, and cigarettes [51]. All of them are associated with increased production of free radicals and oxidative stress [7]. Enhanced formation of ROS and increased oxidative cell damage were observed in both ischemic and hemorrhagic stroke patients. It is believed that oxidative stress is one of the mechanisms causing brain injury in these conditions. Indeed, oxidative stress may be responsible for mitochondrial dysfunction, ceramide accumulation, neuroinflammation, and neuronal apoptosis [48,49,50]. As a result of brain ischemia, ATP synthesis is inhibited and it blocks the activity of the sodium-potassium pump (Na^+^/K^+^-ATPase) causing the inflow of Ca^2^^+^ ions into the cell. As a result, many Ca^2^^+^-dependent enzymes are activated. The activated enzymes generate ROS formation and respond to oxidative damage to cell components. Indeed, enhanced intracellular concentration of Ca^2^^+^ activates phospholipase A2 and cyclooxygenases (COX-1 and COX-2) which not only enhance glutaminergic neurotransmission, but are also responsible for ROS overproduction. Nevertheless, redox imbalance and oxidative stress also occur in hemorrhagic stroke. The sources of free radicals are mitochondrial dysfunction, inflammatory cell activation, and increased activity of NOX and xanthine oxidase (XO) [48,49,50].

Abnormalities in antioxidant systems were previously observed in plasma, serum, and erythrocytes of stroke patients [12,13,50]. Moreover, disturbances in redox homeostasis were also demonstrated in different brain structures of stroke cases [52]. In our study, we were the first to show that the subacute phase of stroke is also associated with alterations in salivary redox status. In the study group, we observed an increase in salivary antioxidant enzymes (↑Px and ↑CAT in NWS and SWS; ↑SOD in NWS) and uric acid (↑UA in NWS and SWS). Generally, this suggests an adaptive response to the enhanced production of free radicals. It is well known that strengthening the antioxidant barrier is the primary defense mechanism against oxidative damage to proteins and lipids.

Most salivary antioxidants are produced outside salivary glands and transported from plasma to the oral cavity. These include both antioxidant enzymes (CAT, SOD, glutathione reductase) and low molecular weight free radical scavengers (UA, GSH) [10,53]. Nevertheless, salivary peroxidase is considered to be the most important enzyme contained in saliva. Although Px represents only 0.01% of salivary proteins, it is the only antioxidant synthesized exclusively by the salivary glands [7,33]. Therefore, the increased activity of Px in NWS and SWS of stroke patients suggests that only the antioxidant barrier of salivary glands is strengthened. UA is also noteworthy because it represents up to 70% of saliva’s antioxidant capacity [10,53]. However, it should be remembered that this compound has also a strong prooxidant effect at high concentrations [54]. Indeed, uric acid can generate free radicals by reacting with peroxynitrite or nitric oxide (NO) to form 6-aminouracyl. Interestingly, previous studies have shown that hyperuricemia worsens the prognosis for patients after a stroke. It also increases the incidence of ischemic stroke in the elderly population [55,56]. However, in our study, salivary UA did not correlate with a decrease in cognitive function or functional independence.

Although we did not directly assess the rate of ROS production, the increased intensity of oxidative processes (in both NWS and SWS) is evidenced by an increase in the TOS level in the study group. TOS characterizes the total amount of oxidants in the analyzed sample [38], while TAC is the resultant ability to scavenge oxygen free radicals [37]. Thus, the increase in the oxidative stress index (TOS to TAC ratio) suggests a shift in the redox balance in favor of the oxidation reaction.

Indeed, in stroke patients, oxidative damage to proteins (↑AGE, ↑AOPP) and lipids (↑LOOH) occurs in both NWS and SWS. Particularly noteworthy is the increase in the protein glycooxidation products which, when combined with a specific receptor (RAGE, receptor for advanced glycation end products), activate the pro-inflammatory transcription factor NFkB and other signaling pathways (e.g., MAP-kinases, NJK, and p21RAS) [9,57]. Thus, the production of cytokines and growth factors (e.g., IL-1, IL-6, TNF-α), the expression of intracellular adhesion molecules (ICAM, intracellular adhesion molecule; VCAM, vascular cell adhesion molecule), and the accumulation/aggregation of oxidized proteins in the cell are increased. Interestingly, AGE and AOPP, by induction of NOX activity, further increase ROS production and exacerbate inflammation [9,57,58].

Redox biomarkers may give particular information that enable the differential diagnosis, prognosis, and staging of systemic diseases and they report on the presence of confounding comorbidities [59]. At present, saliva is seen as ideal, inexpensive, and non-invasive diagnostic fluid compared with blood [11]. The risk of infection of medical professionals and/or patients with some pathogens might be lowered during collection and diagnosis. Moreover, samples can be easily obtained without any discomfort to the patient. The procedure is mostly well tolerated by the elderly, children, and people with disabilities [60].

In the study group, we observed a decrease in reduced glutathione content. Apart from ROS decomposition and regeneration of other antioxidants (e.g., vitamin C and E), GSH facilitates the repair of oxidatively damaged cell components. GSH is also thought to be the most important brain antioxidant [61]. Cerebral GSH maintains a redox balance (as the main cellular thiol buffer) and is involved in the glutaminergic transmission. Therefore, reduction of brain GSH impairs the growth, differentiation, and apoptosis of neurons and glial cells. It is also postulated that disturbances in glutathione metabolism may result in cerebral degeneration and cognitive impairment [9,61]. In this study we showed that GSH assessed in SWS significantly differentiates stroke patients from the control group. The diagnostic usefulness was also confirmed using the ROC analysis. Salivary GSH with 100% specificity and 100% sensitivity differentiates the analyzed groups (AUC = 1.0). The salivary GSH also positively correlates with the decrease in cognitive function in ACE III and dynamic balance abilities in BBS. Interestingly, in our earlier study, we demonstrated the diagnostic usefulness of salivary GSH in differential diagnosis of dementia [22]. This parameter correlated not only with the degree of dementia severity, but also with its blood level. It is therefore necessary to assess the salivary–blood correlation of GSH in stroke patients. Diagnostic usefulness should also be analyzed in a larger number of patients. However, in the multifactorial regression model, we have shown that the variables affecting GSH concentration in SWS are only the presence of stroke and patient’s inability to safely balance during a series of predetermined tasks in BBS. Nevertheless, the GSH level does not depend on gender, age, or salivary secretion.

Stroke patients also suffer from salivary gland dysfunction which manifests itself as a decrease in stimulated salivary flow and total protein content in saliva. It is commonly known that salivary gland secretion is initiated by reflex nerve impulses, which causes the release of neurotransmitters at nerve endings of the salivary glands [62]. However, salivary nuclei receive information from higher centers in addition to the stimuli received via afferent pathways [62,63]. Thus, it is very likely that the salivary nuclei are damaged during the stroke, resulting in a decrease in salivary secretion. During stimulation, up to 60% of saliva is produced by the parotid gland [63]. Therefore, in patients with stroke, dysfunction of mainly parotid glands occurs. The potential role of oxidative stress in salivary gland hypofunction may be indicated by a negative correlation between AGE, AOPP, LOOH content, and stimulated salivary flow. A similar relationship was observed in other oxidative stress-related systemic diseases, such as psoriasis [31], hypertension [18], and chronic kidney disease [64]. Interestingly, the content of protein and lipid oxidation products did not correlate with salivary flow in healthy individuals (data not shown). In the multifactorial regression model, we also showed that the concentration of protein (AGE, AOPP) and lipid (LOOH) oxidation products depends on salivary flow rate. The products of protein and lipid oxidation have, therefore, limited diagnostic value.

The oral health status of the study population was unsatisfactory, with caries prevalence that was 100.00%. This is in accordance with the study by Károlyházy et al. [65]. The authors examined 102 stroke patients; however, they were at least one year after the stroke episode, and they revealed that the oral health status of stroke patients deteriorated in comparison to the sex-matched control group [65]. The results of their study were similar to our findings since they found (AGE, AOPP) and lipid (LOOH) oxidation products depends on salivary flow rate. The products of protein and lipid oxidation have, therefore, limited diagnostic value.

The oral health status of the study population was unsatisfactory, with caries prevalence that was 100.00%. This is in accordance with the study by Károlyházy et al. [65]. The authors examined 102 stroke patients; however, they were at least one year after the stroke episode, and they revealed that the oral health status of stroke patients deteriorated in comparison to the sex-matched control group [65]. The results of their study were similar to our findings since they found that stroke patients had significantly more missing teeth and fewer filled teeth than did the healthy controls. In addition, in our research a number of patients had carious teeth or had caries complications that were potential focuses of infections. Interestingly, Miyatani et al. suggested that *Streptococcus mutans* with Cnm collagen-binding ability is involved in cerebral microbleeds [51]. The Cnm protein is situated on the cell surface of the bacteria and easily binds the collagen of tooth dentin. Cerebral vascular endothelial cells, in cerebral hemorrhage, are destroyed and collagen is exposed. Such a situation in connection with hemostasis by platelets is a vital defense mechanism against cerebral hemorrhage. However, while *Streptococcus mutans* possessing the Cnm gene that encodes the Cnm protein is transferred from oral cavity into the circulation, the bacteria might cling to and damage the vascular endothelium, promoting cerebral hemorrhage [51,66]. Additionally, cofactors such as redox imbalance and AGE formation can also play an important role in the onset of dental caries and periodontal disease [67,68].

Needless to say, a close interdisciplinary collaboration between different specialists, including, e.g., neurologists, rehabilitation staff, general physicians, and dentists is recommended. In addition, oral health medical staff need to be involved not only in treating such individuals, but also in prevention and counseling [69]. It must be emphasized that maintaining acceptable oral hygiene in stroke survivors who are affected by paresis of various degrees could be remarkably challenging. Therefore, it needs to be emphasized that manual dexterity is essential for toothbrushing to maintain satisfactory oral hygiene.

Attention should also be paid to the limitations of our study. We evaluated only selected antioxidants and biomarkers of oxidative stress, so we cannot fully characterize the redox homeostasis of stroke patients. It is advisable to assess the glutathione and LOOH concentrations using chromatographic methods, which could increase the accuracy and repeatability of results. Moreover, we determined the redox status exclusively in saliva. In subsequent studies, it will be necessary to assess the saliva–blood correlation of redox biomarkers and assess their diagnostic utility in a larger group of patients. Additionally, saliva samples were taken from patients in the shortest possible time, i.e., in the early subacute phase of the stroke. The material collection immediately after the stroke was impossible due to greater or lesser physical and cognitive deficits of patients. It was also impossible to obtain informed and written consent from all patients. Moreover, we cannot exclude the influence of accompanying diseases on the evaluated redox biomarkers. However, the present study also has some strengths. The individuals were properly selected from a population of stroke sufferers. Firstly, most participants followed the same diet, except for the individuals who suffered from diabetes mellitus. Moreover, sampling of the saliva was carried out under the same conditions, during the same season of the year, and processing, handling, storage, and analysis techniques were uniform. Finally, two samples of saliva from each patient, i.e., stimulated and unstimulated, were obtained.

To summarize, the present research revealed that in subacute stroke there occur redox imbalances and oxidative damage to proteins and lipids in non-stimulated and stimulated saliva. Thus, this biofluid seems to be promising diagnostic material. Moreover, stroke patients also suffer from salivary gland dysfunction. Therefore, they should be regularly supervised by a dentist who needs to closely cooperate with other medical professionals who treat stroke sufferers to provide them with comprehensive care.

## 5. Conclusions

In the saliva of patients in the subacute phase of stroke, the enzymatic and non-enzymatic systems are disturbed and oxidative damage to proteins and lipids is increased.

Biomarkers from saliva of stroke sufferers seem to be a promising diagnostic tool (especially reduced glutathione). However, there is a need to continue the research in a larger population of patients.

The subacute phase of stroke is also associated with salivary gland dysfunction (mainly parotid glands).

## Figures and Tables

**Table 1:** Clinical characteristics of the study and control groups.

Patients Characteristics		Control *n* = 30	Study *n* = 30	*p*-Value
Sex	male *n* (%)	18 (60.00)	18 (60.00)	ns
	female *n* (%)	12 (40.00)	12 (40.00)	
Age in years	(mean ± SD)	61.87 ± 11.81	61.87 ± 11.81	ns
Education	primary *n* (%)	2 (6.67)	3 (10.00)	ns
	vocational *n* (%)	13 (43.33)	12 (40.00)	ns
	secondary *n* (%)	8 (26.67)	7 (23.33)	ns
	university *n* (%)	7 (23.33)	8 (26.67)	ns
Place of residence	urban centre *n* (%)	11 (36.67)	8 (26.67)	ns
	small town *n* (%)	7 (23.33)	11 (36.67)	ns
	rural area or small village *n* (%)	12 (40.00)	11 (36.67)	ns
Houshold member(s)	with family member *n* (%)	16 (53.33)	21 (70.00)	ns
	none *n* (%)	14 (46.67)	9 (30.00)	ns
Type of stroke	hemorrhagic *n* (%)	-	6 (20.00)	nd
	ischemic *n* (%)	-	23 (76.67)	nd
	ischemic → hemorrhagic *n* (%)	-	1 (3.33)	nd
Time from stroke incident to dental examination and saliva sampling	(mean ± SD)	-	46.78 ± 4.71	nd
Numbers of strokes in the patient’s life	1	-	26 (86.67)	nd
	2	-	4 (13.33)	nd
Cognitive and physical functional status	ACE III (mean ± SD)	97.07 ± 1.26	68.80 ± 21.79	<0.0001
	BI (mean ± SD)	20 ± 0	11.10 ± 3.85	<0.0001
	FIM (mean ± SD)	125.2 ± 0.66	86.73 ± 31.14	<0.0001
	BBS (mean ± SD)	55.47 ± 0.5	31.03 ± 16.50	<0.0001
Other disorders	hypertension *n* (%)	20 (66.67)	22 (73.33)	ns
	diabetes *n* (%)	8 (26.67)	8 (26.67)	ns
	epilepsy *n* (%)	3 (10.00)	3 (10.00)	ns
	arteriosclerosis *n* (%)	6 (20)	7 (23.33)	ns
	gout *n* (%)	1 (3.33)	2 (6.67)	ns
	limb thrombosis *n* (%)	2 (6.67)	2 (6.67)	ns
	atrial fibrillation *n* (%)	3 (10.00)	4 (13.33)	ns
Medications	<5 drugs/day *n* (%)	11 (36.67)	12 (40.00)	ns
	≥5 drugs/day *n* (%)	16 (53.33)	18 (60.00)	ns

ACE III: Addenbrooke’s Cognitive Examination III; BI: Barthel Index; FIM: functional independence measure; BBS: the Berg Balance Scale; ns: non-significant; nd—no data.

**Table 2:** Dental characteristics of study group compared to the controls.

	Control *n* = 30	Study *n* = 30	*p*-Value
Caries prevalence (%), i.e., percentage of individuals with DMFT > 0	100.00	100.00	ns
DT	3.2 ± 1.2	2.50 ± 3.15	ns
MT	10.5 ± 5.8	17.50 ± 11.41	0.0038
FT	8 ± 2.1	3.13 ± 4.97	<0.0001
DMFT	25 ± 5.3	23.13 ± 7.32	ns
<5	0 (0.00)	0 (0.00)	ns
5–9	3 (10.00)	1 (3.33)	ns
10–14	1 (3.33)	3 (10.00)	ns
15–19	5 (16.67)	5 (16.67)	ns
20–25	2 (6.66)	6 (20.00)	ns
>25	19 (63.33)	15 (50.00)	ns
GI (six completely edentulous patients were excluded from calculations, i.e., *n* = 24)	0.74 ± 0.2	0.83 ± 0.86	ns
Number of patients without gingivitis *n* (%)	15 (50.00)	8 (33.33)	ns
Number of patients with mild gingivitis *n* (%)	8 (33.33)	9 (37.50)	ns
Number of patients with moderate gingivitis *n* (%)	4 (16.67)	4 (16.67)	ns
Number of patients with severe gingivitis *n* (%)	3 (12.50)	3 (12.50)	ns
PlI (six completely edentulous patients were excluded from calculations, i.e., *n* = 24)	1.18 ± 0.58	1.29 ± 0.98	ns
Number of patients with excellent hygiene *n* (%)	15 (50.00)	4 (16.67)	ns
Number of patients with good hygiene *n* (%)	9 (37.50)	9 (37.50)	ns
Number of patients with satisfactory hygiene *n* (%)	5 (20.83)	6 (25.00)	ns
Number of patients with unsatisfactory hygiene *n* (%)	1 (3.33)	5 (20.83)	<0.0001

Abbreviations: DMFT—caries severity index; that is, a sum of decayed teeth (DT), teeth missing due to carious process (MT), and teeth filled because of caries (FT); *n*: number of patients; GI: gingival index; PlI: plaque index; ns: non-significant.

**Table 3:** Salivary gland function and dental characteristics of stroke survivors compared to the controls.

	Control *n* = 30	Study *n* = 30	*p*-Value
NWS flow rate (mL/min)	0.33 ± 0.09	0.42 ± 0.25	ns
SWS flow rate (mL/min)	0.91 ± 0.26	0.68 ± 0.32	0.0036
TP NWS (μg/mL)	1213 ± 196.4	1159 ± 270.1	ns
TP SWS (μg/mL)	1307 ± 170.4	968.2 ± 247	<0.0001

NWS: non-stimulated whole saliva; SWS: stimulated whole saliva; TP: total protein concentration; ns: non-significant.

**Table 4:** **Multifactorial regression of salivary redox biomarkers in all enrolled patients.** Age, ACE III: Addenbrooke’s Cognitive Examination III, BI: Barthel Index, FIM: functional independence measure, BBS: the Berg Balance Scale, NWS flow, and SWS flow were included as continuous variables; *—*p* < 0.05. For stoke, controls served as a reference, and for gender—females. Regression parameters were reported along with 95% confidence intervals.

Dependent Variables	Independent Variables								
	Stroke	Gender: Male	Age	ACE III	BI	FIM	BBS	NWS Flow	SWS Flow
**Px NWS**	0.019 (−0.011–0.049), *p* = 0.218	0.003 (−0.009–0.016), *p* = 0.6	0 (0–0.001), *p* = 0.191	0 (−0.001–0), *p* = 0.143	−0.002 (−0.006–0.001), *p* = 0.228	0 (−0.001–0), *p* = 0.297	0.001 (0–0.002), *p* = 0.027 *	−0.007 (−0.04–0.027), *p* = 0.705	0.02 (−0.001–0.042), *p* = 0.07
**Px SWS**	0.042 (0.008–0.077), *p* = 0.021 *	−0.002 (−0.016–0.012), *p* = 0.784	0 (−0.001–0.001), *p* = 0.822	0 (−0.001–0), *p* = 0.149	0 (−0.004–0.005), *p* = 0.829	−0.001 (−0.002–0), *p* = 0.077	0.001 (0–0.002), *p* = 0.106	0.033 (−0.006–0.072), *p* = 0.103	0.013 (−0.012–0.038), *p* = 0.321
**CAT NWS**	0.119 (−0.404–0.641), *p* = 0.658	−0.071 (−0.286–0.143), *p* = 0.518	−0.006 (−0.015–0.003), *p* = 0.204	0.003 (−0.005–0.011), *p* = 0.482	−0.062 (−0.125–0.001), *p* = 0.058	−0.011 (−0.023–0.001), *p* = 0.074	0.025 (0.007–0.042), *p* = 0.008 *	−0.166 (−0.758–0.426), *p* = 0.586	0.038 (−0.339–0.415), *p* = 0.844
**CAT SWS**	−0.236 (−0.755–0.283), *p* = 0.377	−0.01 (−0.223–0.204), *p* = 0.928	−0.009 (−0.018–0), *p* = 0.049 *	−0.006 (−0.014–0.001), *p* = 0.109	−0.029 (−0.092–0.033), *p* = 0.364	0.003 (−0.008–0.015), *p* = 0.58	−0.015 (−0.032–0.003), *p* = 0.101	0.222 (−0.366–0.811), *p* = 0.463	−0.005 (−0.38–0.369), *p* = 0.979
**SOD NWS**	4.192 (−1.469–9.852), *p* = 0.153	−1.388 (−3.717–0.94), *p* = 0.248	0.042 (−0.053–0.137), *p* = 0.39	0.02 (−0.065–0.105), *p* = 0.648	0.262 (−0.421–0.945), *p* = 0.455	−0.084 (−0.213–0.045), *p* = 0.209	0.11 (−0.079–0.299), *p* = 0.26	−0.485 (−6.902–5.933), *p* = 0.883	−1.399 (−5.482–2.684), *p* = 0.505
**SOD SWS**	−4.672 (−13.971–4.627), *p* = 0.329	3.338 (−0.487–7.164), *p* = 0.093	0.114 (−0.042–0.27), *p* = 0.157	0.02 (−0.119–0.159), *p* = 0.776	−0.537 (−1.658–0.584), *p* = 0.352	0.089 (−0.123–0.301), *p* = 0.415	−0.044 (−0.354–0.266), *p* = 0.784	−6.139 (−16.68–4.403), *p* = 0.259	0.227 (−6.479–6.934), *p* = 0.947
**UA NWS**	5.399 (−12.355–23.153), *p* = 0.554	1.604 (−5.7–8.908), *p* = 0.669	0.112 (−0.186–0.409), *p* = 0.465	−0.124 (−0.39–0.142), *p* = 0.364	−0.15 (−2.291–1.99), *p* = 0.891	0.162 (−0.243–0.567), *p* = 0.437	−0.285 (−0.877–0.307), *p* = 0.349	3.155 (−16.972–23.281), *p* = 0.76	−7.858 (−20.662–4.946), *p* = 0.235
**UA SWS**	−5.194 (−35.68–25.292), *p* = 0.74	7.91 (−4.632–20.452), *p* = 0.222	−0.117 (−0.628–0.394), *p* = 0.655	−0.062 (−0.518–0.395), *p* = 0.792	−3.226 (−6.902–0.449), *p* = 0.092	0.069 (−0.627–0.765), *p* = 0.846	0.249 (−0.767–1.266), *p* = 0.633	7.454 (−27.106–42.014), *p* = 0.674	6.264 (−15.722–28.251), *p* = 0.579
**GSH NWS**	−0.276 (−4.439–3.887), *p* = 0.897	−0.073 (−1.786–1.64), *p* = 0.934	−0.027 (−0.097–0.043), *p* = 0.456	−0.064 (−0.126–0.001), *p* = 0.05	0.189 (−0.313–0.691), *p* = 0.463	0.04 (−0.055–0.135), *p* = 0.41	−0.078 (−0.217–0.06), *p* = 0.273	3.333 (−1.387–8.053), *p* = 0.172	−0.383 (−3.386–2.619), *p* = 0.803
**GSH SWS**	−16.637 (−24.716–8.557), *p* < 0.001 *	−0.809 (−4.133–2.515), *p* = 0.635	0.061 (−0.074–0.197), *p* = 0.38	0.301 (0.18–0.422), *p* < 0.001 *	0.278 (−0.696–1.252), *p* = 0.579	−0.004 (−0.188–0.181), *p* = 0.967	−0.049 (−0.318–0.221), *p* = 0.724	−2.611 (−11.771–6.548), *p* = 0.579	−0.747 (−6.574–5.08), *p* = 0.803
**TAC NWS**	0.551 (−0.82–1.923), *p* = 0.434	0.08 (−0.484–0.644), *p* = 0.783	0.011 (−0.012–0.034), *p* = 0.334	−0.004 (−0.025–0.016), *p* = 0.684	−0.083 (−0.248–0.082), *p* = 0.33	−0.013 (−0.045–0.018), *p* = 0.406	0.044 (−0.001–0.09), *p* = 0.062	−0.903 (−2.457–0.652), *p* = 0.261	1.099 (0.11–2.088), *p* = 0.034 *
**TAC SWS**	−0.808 (−2.428–0.812), *p* = 0.333	−0.772 (−1.439–0.106), *p* = 0.028 *	−0.002 (−0.029–0.025), *p* = 0.903	0.005 (−0.019–0.029), *p* = 0.694	−0.173 (−0.368–0.022), *p* = 0.089	0.043 (0.006–0.08), *p* = 0.027 *	−0.076 (−0.13–0.022), *p* = 0.008 *	0.113 (−1.724–1.949), *p* = 0.905	0.975 (−0.193–2.144), *p* = 0.108
**TOS NWS**	21.886 (−44.111–87.883), *p* = 0.519	−2.844 (−29.996–24.307), *p* = 0.838	0.144 (−0.963–1.25), *p* = 0.8	−0.19 (−1.178–0.798), *p* = 0.708	−2.317 (−10.275–5.641), *p* = 0.571	−0.826 (−2.333–0.68), *p* = 0.287	1.356 (−0.844–3.557), *p* = 0.233	−20.546 (−95.364–54.272), *p* = 0.593	25.999 (−21.598–73.596), *p* = 0.289
**TOS SWS**	30.97 (−126.85–188.789), *p* = 0.702	34.294 (−30.633–99.222), *p* = 0.306	1.398 (−1.248–4.045), *p* = 0.305	0.451 (−1.911–2.813), *p* = 0.71	−16.533 (−35.562–2.496), *p* = 0.095	−0.255 (−3.858–3.347), *p* = 0.89	0.171 (−5.092–5.434), *p* = 0.95	103.489 (−75.423–282.401), *p* = 0.262	−106.848 (−220.666–6.971), *p* = 0.072
**OSI NWS**	−6.811 (−53.35–39.727), *p* = 0.775	−0.109 (−19.255–19.037), *p* = 0.991	−0.219 (−1–0.561), *p* = 0.584	−0.16 (−0.856–0.537), *p* = 0.655	0.072 (−5.539–5.684), *p* = 0.98	0.092 (−0.971–1.154), *p* = 0.866	−0.536 (−2.088–1.015), *p* = 0.501	23.291 (−29.467–76.049), *p* = 0.391	−14.16 (−47.723–19.404), *p* = 0.412
**OSI SWS**	32.132 (−20.171–84.434), *p* = 0.234	27.376 (5.858–48.893), *p* = 0.016 *	0.294 (−0.583–1.171), *p* = 0.514	0.052 (−0.731–0.834), *p* = 0.898	−1.561 (−7.867–4.745), *p* = 0.63	−0.932 (−2.126–0.262), *p* = 0.132	1.752 (0.008–3.497), *p* = 0.054	43.924 (−15.369–103.216), *p* = 0.153	−43.958 (−81.678–6.238), *p* = 0.027 *
**AGE NWS**	2.145 (−124.129–128.42), *p* = 0.974	−16.176 (−68.126–35.774), *p* = 0.544	−0.115 (−2.232–2.002), *p* = 0.916	−1.588 (−3.478–0.301), *p* = 0.106	−6.18 (−21.406–9.045), *p* = 0.43	0.936 (−1.946–3.818), *p* = 0.527	−1.245 (−5.455–2.966), *p* = 0.565	118.067 (−25.084–261.219), *p* = 0.112	38.166 (−52.903–129.235), *p* = 0.415
**AGE SWS**	149.753 (18.494–281.011), *p* = 0.03 *	6.864 (−47.136–60.865), *p* = 0.804	0.414 (−1.787–2.614), *p* = 0.714	0.117 (−1.847–2.081), *p* = 0.907	14.242 (−1.584–30.069), *p* = 0.084	−2.386 (−5.382–0.61), *p* = 0.125	0.918 (−3.459–5.295), *p* = 0.683	−119.668 (−268.47–29.133), *p* = 0.121	−122.463 (−217.127–27.8), *p* = 0.014 *
**AOPP NWS**	46.735 (16.635–76.835), *p* = 0.004 *	2.443 (−9.94–14.826), *p* = 0.701	0.026 (−0.478–0.531), *p* = 0.919	0.14 (−0.31–0.59), *p* = 0.545	3.003 (−0.626–6.632), *p* = 0.111	−0.574 (−1.261–0.113), *p* = 0.108	−0.105 (−1.109–0.898), *p* = 0.838	−16.026 (−50.148–18.097), *p* = 0.362	−11.351 (−33.059–10.357), *p* = 0.31
**AOPP SWS**	47.369 (29.289–65.45), *p* < 0.001 *	1.939 (−5.499–9.378), *p* = 0.612	−0.024 (−0.327–0.279), *p* = 0.879	0.058 (−0.213–0.328), *p* = 0.677	1.815 (−0.365–3.995), *p* = 0.109	−0.043 (−0.456–0.37), *p* = 0.839	−0.414 (−1.017–0.188), *p* = 0.184	−30.24 (−50.737–9.743), *p* = 0.006 *	−23.316 (−36.356–10.277), *p* = 0.001 *
**LOOH NWS**	163.531 (−17.81–344.873), *p* = 0.083	−14.451 (−89.056–60.153), *p* = 0.706	0.488 (−2.552–3.529), *p* = 0.754	−0.917 (−3.631–1.796), *p* = 0.511	1.999 (−19.866–23.864), *p* = 0.858	1.785 (−2.354–5.924), *p* = 0.402	−3.443 (−9.49–2.604), *p* = 0.27	41.144 (−164.434–246.722), *p* = 0.697	−7.547 (−138.33–123.235), *p* = 0.91
**LOOH SWS**	300.808 (128.923–472.693), *p* = 0.001 *	27.154 (−43.56–97.868), *p* = 0.455	1.502 (−1.38–4.384), *p* = 0.312	−1.306 (−3.878–1.267), *p* = 0.325	−11.257 (−31.982–9.468), *p* = 0.292	1.064 (−2.859–4.987), *p* = 0.597	1.491 (−4.241–7.223), *p* = 0.612	−89.548 (−284.405–105.31), *p* = 0.372	−238.519 (−362.482–114.556), *p* < 0.001 *

**Table 5:** Correlations between clinical parameters, redox biomarkers, and salivary flow in stroke patients.

	ACE III	BI	FIM	BBS	NWS Flow	SWS Flow	ACE III	BI	FIM	BBS	NWS Flow	SWS Flow
	*r*-value						*p*-value					
Px NWS	−0.13	−0.26	−0.15	0.05	−0.15	0.27	0.52	0.17	0.44	0.80	0.44	0.15
CAT NWS	0.04	−0.43	−0.28	−0.03	−0.07	0.07	0.83	0.02	0.15	0.90	0.71	0.73
SOD NWS	0.04	0.04	−0.09	0.05	−0.03	−0.34	0.85	0.82	0.63	0.80	0.89	0.06
UA NWS	−0.23	0.06	−0.02	−0.10	−0.08	−0.38	0.22	0.78	0.94	0.60	0.67	0.04
GSH NWS	−0.34	0.16	−0.01	−0.12	0.27	0.05	0.07	0.40	0.95	0.55	0.16	0.80
TAC NWS	0.01	−0.17	−0.02	0.13	−0.18	0.21	0.95	0.39	0.93	0.51	0.35	0.27
TOS NWS	−0.13	−0.31	−0.27	−0.13	−0.02	0.12	0.49	0.10	0.15	0.51	0.90	0.52
OSI NWS	−0.14	−0.08	−0.16	−0.19	0.16	−0.10	0.46	0.68	0.41	0.32	0.41	0.58
AGE NWS	−0.23	−0.16	−0.17	−0.23	0.19	0.16	0.23	0.40	0.39	0.23	0.31	0.40
AOPP NWS	−0.16	−0.09	−0.32	−0.27	−0.16	−0.29	0.41	0.63	0.09	0.15	0.41	0.12
LOOH NWS	−0.08	0.07	0.00	−0.11	0.07	0.19	0.68	0.73	0.99	0.56	0.73	0.32
Px SWS	−0.24	−0.21	−0.29	−0.18	0.23	0.11	0.22	0.28	0.12	0.34	0.23	0.58
CAT SWS	−0.36	−0.35	−0.41	−0.45	0.10	0.05	0.05	0.07	0.03	0.01	0.61	0.81
SOD SWS	0.08	−0.02	0.13	0.14	−0.31	−0.11	0.70	0.93	0.50	0.48	0.10	0.58
UA SWS	−0.02	−0.29	−0.12	−0.04	0.02	0.19	0.91	0.12	0.54	0.83	0.94	0.32
GSH SWS	0.84	0.22	0.35	0.25	0.17	0.32	0.00	0.25	0.05	0.19	0.38	0.08
TAC SWS	0.14	−0.24	−0.14	−0.30	0.20	0.43	0.46	0.21	0.49	0.11	0.29	0.02
TOS SWS	−0.13	−0.40	−0.33	−0.30	0.04	−0.10	0.52	0.03	0.09	0.12	0.85	0.60
OSI SWS	−0.13	−0.12	−0.11	0.06	−0.02	−0.27	0.50	0.55	0.58	0.78	0.90	0.16
AGE SWS	−0.30	0.09	−0.23	−0.12	−0.34	−0.79	0.11	0.63	0.24	0.55	0.06	0.00
AOPP SWS	−0.26	0.16	−0.07	−0.10	−0.47	−0.76	0.17	0.41	0.74	0.60	0.01	0.00
LOOH SWS	−0.25	0.04	0.05	0.13	−0.43	−0.77	0.20	0.84	0.80	0.52	0.02	0.00

ACE III: Addenbrooke’s Cognitive Examination III; BI: Barthel Index; FIM: functional independence measure; BBS: Berg Balance Scale; CAT: catalase; SOD: superoxide dismutase-1; UA: uric acid; GSH: reduced glutathione; TAC: total antioxidant capacity; TOS: total oxidant status; OSI: oxidative stress index; AGE: advanced glycation end products; AOPP: advanced oxidation protein products; LOOH: lipid hydro-peroxides.

**Table 6:** ROC analysis of antioxidant defense, redox status, and oxidative damage products in non-stimulated saliva of stroke patients compared to the controls.

	AUC	95% Confidence Interval (CI)	Cut Off	Sensitivity%	95% CI	Specificity%	95% CI
Px	0.8078	0.6880 to 0.9276	>0.03932	76.67	59.07% to 88.21%	73.33	55.55% to 85.82%
CAT	0.8122	0.7003 to 0.9241	>0.3415	76.67	59.07% to 88.21%	70	52.12% to 83.34%
SOD	0.6189	0.4753 to 0.7625	>12.88	63.33	45.51% to 78.13%	53.33	36.14% to 69.77%
UA	0.7611	0.6403 to 0.8819	>55.47	70	52.12% to 83.34%	63.33	45.51% to 78.13%
GSH	0.5367	0.3891 to 0.6843	>13.22	50	33.15% to 66.85%	50	33.15% to 66.85%
TAC	0.6144	0.4645 to 0.7644	>3.036	56.67	39.20% to 72.62%	56.67	39.20% to 72.62%
TOS	0.7222	0.5938 to 0.8507	>189.0	63.33	45.51% to 78.13%	60	42.32% to 75.41%
AGE	0.8078	0.6889 to 0.9267	>361.7	80	62.69% to 90.49%	76.67	59.07% to 88.21%
AOPP	0.8711	0.7653 to 0.9770	>32.58	83.33	66.44% to 92.66%	76.67	59.07% to 88.21%
LOOH	0.8567	0.7602 to 0.9531	>359.5	86.67	70.32% to 94.69%	80	62.69% to 90.49%

**Table 7:** ROC analysis of antioxidant defense, redox status, and oxidative damage products in stimulated saliva of stroke patients compared to the controls.

	AUC	95% Confidence Interval (CI)	Cut Off	Sensitivity%	95% CI	Specificity%	95% CI
Px	0.9478	0.8916 to 1.000	>0.04009	90	74.38% to 96.54%	90	74.38% to 96.54%
CAT	0.7578	0.6194 to 0.8962	>0.5207	76.67	59.07% to 88.21%	66.67	48.78% to 80.77%
SOD	0.6422	0.4994 to 0.7850	<29.16	63.33	45.51% to 78.13%	60	42.32% to 75.41%
UA	0.6533	0.5101 to 0.7966	>57.45	53.33	36.14% to 69.77%	60	42.32% to 75.41%
GSH	1	1.000 to 1.000	<42.71	100	88.65% to 100.0%	100	88.65% to 100.0%
TAC	0.5344	0.3747 to 0.6942	>3.893	56.67	39.20% to 72.62%	53.33	36.14% to 69.77%
TOS	0.8822	0.7934 to 0.9711	>286.4	86.67	70.32% to 94.69%	83.33	66.44% to 92.66%
AGE	0.7522	0.6300 to 0.8744	>302.2	73.33	55.55% to 85.82%	66.67	48.78% to 80.77%
AOPP	0.9911	0.9763 to 1.000	>54.50	96.67	83.33% to 99.83%	93.33	78.68% to 98.82%
LOOH	0.9833	0.9604 to 1.000	>548.2	93.33	78.68% to 98.82%	90	74.38% to 96.54%

## Supplementary Materials

The following are available online at https://www.mdpi.com/2077-0383/9/7/2252/s1. Table S1: Salivary redox biomarkers in patients with ischemic and hemorrhagic stroke and control. Table S2: Multifactorial regression of salivary redox biomarkers in patients with ischemic and hemorrhagic stroke and control.
