# Supplementary material for: Comprehensive Evaluation of the Oral Health Status, Salivary Gland Function, and Oxidative Stress in the Saliva of Patients with Subacute Phase of Stroke: A Case-Control Study
Source: J Clin Med. 2020 Jul 15;9(7):2252. doi: 10.3390/jcm9072252 (PMC7408698; doi:10.3390/jcm9072252)
Supplement: Supplementary file 1 [file jcm-09-02252-s001.pdf]

Table 1. Salivary redox biomarkers in patients with ischemic and hemorrhagic stroke and control.

**Parametr**		**Group**			***p***
		**Control – A (*N* = 30)**	**Ischemic stroke - B (*N* = 24)**	**Hemorrhagic stroke - C (*N* = 6)**	
**AGE NWS**	**Mean ± SD**	422.33 ± 113.46	331.38 ± 72.66	295.55 ± 32.85	$p = 0.001$ *, A>B,C
AGE SWS	Mean ± SD	361.63 ± 104.85	243.02 ± 112.12	306.54 ± 95.29	$p = 0.001$ *, A>B
AOPP NWS	Mean ± SD	68.95 ± 31.15	26.03 ± 7.36	30.39 ± 11.39	$p < 0.001$ *, A>C,B
AOPP SWS	Mean ± SD	83.26 ± 20.99	39.33 ± 9.25	40.1 ± 12.67	$p < 0.001$ *, A>C,B
CAT NWS	Mean ± SD	0.7 ± 0.6	0.27 ± 0.1	0.33 ± 0.11	$p = 0.002$ *, A>B
CAT SWS	Mean ± SD	0.92 ± 0.59	0.47 ± 0.15	0.41 ± 0.11	$p = 0.001$ *, A>B,C
GSH NWS	Mean ± SD	13.28 ± 3.18	12.7 ± 3.29	13.27 ± 2.12	$p = 0.782$,
GSH SWS	Mean ± SD	29.9 ± 7.72	56.88 ± 6.93	53.1 ± 5.37	$p < 0.001$ *, B,C>A
LOOH NWS	Mean ± SD	490.6 ± 133.83	320.4 ± 122.25	221.63 ± 66.96	$p < 0.001$ *, A>B,C
LOOH SWS	Mean ± SD	820.67 ± 182.33	405.85 ± 91.68	439.95 ± 124.31	$p < 0.001$ *, A>C,B
NWS flow	Mean ± SD	0.42 ± 0.25	0.34 ± 0.09	0.29 ± 0.08	$p = 0.166$,
OSI NWS	Mean ± SD	73.34 ± 41.19	60.68 ± 21.83	64.32 ± 20.45	$p = 0.371$,
OSI SWS	Mean ± SD	104.19 ± 50.78	51.77 ± 31.62	49.22 ± 14.78	$p < 0.001$ *, A>B,C
Px NWS	Mean ± SD	0.06 ± 0.03	0.03 ± 0.01	0.03 ± 0.01	$p < 0.001$ *, A>C,B
Px SWS	Mean ± SD	0.08 ± 0.04	0.03 ± 0.01	0.03 ± 0.01	$p < 0.001$ *, A>C,B
SOD NWS	Mean ± SD	14.67 ± 3.67	11.78 ± 4.61	15.52 ± 2.36	$p = 0.019$ *, C,A>B
SOD SWS	Mean ± SD	26.97 ± 7.87	30.2 ± 5.57	30.93 ± 6.78	$p = 0.174$,
SWS flow	Mean ± SD	0.68 ± 0.32	0.88 ± 0.26	1.02 ± 0.29	$p = 0.008$ *, C,B>A
TAC NWS	Mean ± SD	3.5 ± 1.36	2.94 ± 0.62	3.08 ± 0.25	$p = 0.15$,
TAC SWS	Mean ± SD	4.41 ± 1.85	3.82 ± 0.54	3.7 ± 0.46	$p = 0.225$,
TOS NWS	Mean ± SD	215.33 ± 51.16	170.31 ± 42.44	195 ± 47.2	$p = 0.004$ *, A>B
TOS SWS	Mean ± SD	400.43 ± 139.39	192.46 ± 106.86	180.43 ± 56.05	$p < 0.001$ *, A>B,C
UA NWS	Mean ± SD	65.7 ± 14.73	51.14 ± 9.95	59.37 ± 8.66	$p < 0.001$ *, A>B
UA SWS	Mean ± SD	67.79 ± 28.73	53.07 ± 12.45	44.44 ± 16.09	$p = 0.017$ *, A>B,C

p - ANOVA + post-hoc analysis (Fishers's LSD test). * statistically significant ($p < 0.05$)

Table 2. Multifactorial regression of salivary redox biomarkers in patients with ischemic and hemorrhagic stroke and control.

Parameter	Hemorrhagic stroke	Ischemic stroke	Gender: Male	Age [yrs]	ACE III	ADL	FIM	Berg	NWS flow	SWS flow
AGE NWS	-14.968 (-149.991-120.055), p=0.829	19.598 (-115.741-154.938), p=0.778	-15.31 (-67.559-36.939), p=0.568	-0.173 (-2.307-1.96), p=0.874	-1.557 (-3.458-0.343), p=0.115	-5.332 (-20.8-10.137), p=0.502	0.915 (-1.982-3.811), p=0.539	-1.048 (-5.312-3.215), p=0.632	130.891 (-17.048-278.83), p=0.089	33.776 (-58.491-126.042), p=0.476
AGE SWS	170.765 (30.711-310.818), p=0.021 *	128.323 (-12.059-268.705), p=0.079	5.801 (-48.395-59.997), p=0.835	0.485 (-1.727-2.698), p=0.669	0.079 (-1.892-2.05), p=0.938	13.2 (-2.845-29.245), p=0.113	-2.36 (-5.364-0.645), p=0.13	0.677 (-3.746-5.1), p=0.765	-135.414 (-288.865-18.037), p=0.09	-117.073 (-212.777--21.369), p=0.02 *
AOPP NWS	49.203 (16.909-81.498), p=0.004 *	44.218 (11.848-76.588), p=0.01 *	2.318 (-10.179-14.815), p=0.718	0.035 (-0.475-0.545), p=0.894	0.136 (-0.319-0.59), p=0.561	2.88 (-0.819-6.58), p=0.133	-0.571 (-1.264-0.122), p=0.112	-0.134 (-1.153-0.886), p=0.799	-17.875 (-53.259-17.508), p=0.327	-10.718 (-32.786-11.35), p=0.346
AOPP SWS	56.203 (38.161-74.245), p<0.001 *	38.359 (20.275-56.443), p<0.001 *	1.492 (-5.489-8.474), p=0.677	0.007 (-0.279-0.292), p=0.964	0.042 (-0.212-0.296), p=0.748	1.377 (-0.689-3.444), p=0.198	-0.032 (-0.419-0.355), p=0.872	-0.516 (-1.085-0.054), p=0.082	-36.86 (-56.627--17.092), p=0.001 *	-21.05 (-33.378--8.721), p=0.002 *
CAT NWS	0.018 (-0.537-0.573), p=0.95	0.221 (-0.335-0.778), p=0.44	-0.066 (-0.281-0.149), p=0.548	-0.006 (-0.015-0.003), p=0.18	0.003 (-0.005-0.011), p=0.454	-0.057 (-0.121-0.006), p=0.083	-0.011 (-0.023-0.001), p=0.071	0.026 (0.008-0.043), p=0.006 *	-0.09 (-0.699-0.518), p=0.772	0.012 (-0.367-0.392), p=0.95
CAT SWS	-0.424 (-0.961-0.112), p=0.127	-0.044 (-0.582-0.493), p=0.872	0 (-0.208-0.207), p=0.997	-0.01 (-0.018--0.001), p=0.031 *	-0.006 (-0.014-0.001), p=0.118	-0.02 (-0.081-0.042), p=0.528	0.003 (-0.008-0.015), p=0.596	-0.013 (-0.03-0.004), p=0.152	0.363 (-0.225-0.951), p=0.232	-0.053 (-0.42-0.313), p=0.776
GSH NWS	-0.11 (-4.584-4.363), p=0.962	-0.445 (-4.929-4.039), p=0.846	-0.081 (-1.813-1.65), p=0.927	-0.026 (-0.097-0.045), p=0.472	-0.064 (-0.127--0.001), p=0.052	0.181 (-0.331-0.694), p=0.492	0.04 (-0.056-0.136), p=0.413	-0.08 (-0.222-0.061), p=0.27	3.208 (-1.693-8.11), p=0.206	-0.341 (-3.398-2.716), p=0.828
GSH SWS	-18.162 (-26.758--9.567), p<0.001 *	-15.081 (-23.697--6.466), p=0.001 *	-0.732 (-4.058-2.594), p=0.668	0.056 (-0.08-0.192), p=0.422	0.303 (0.182-0.424), p<0.001 *	0.354 (-0.631-1.338), p=0.485	-0.006 (-0.19-0.179), p=0.952	-0.031 (-0.303-0.24), p=0.822	-1.468 (-10.886-7.949), p=0.761	-1.138 (-7.012-4.735), p=0.706
LOOH NWS	187.958 (-5.959-381.876), p=0.063	138.619 (-55.754-332.991), p=0.168	-15.688 (-90.727-59.352), p=0.684	0.572 (-2.492-3.635), p=0.716	-0.961 (-3.691-1.768), p=0.493	0.788 (-21.427-23.004), p=0.945	1.815 (-2.345-5.975), p=0.397	-3.723 (-9.847-2.401), p=0.239	22.84 (-189.628-235.307), p=0.834	-1.281 (-133.792-131.231), p=0.985
LOOH SWS	339.484 (157.436-521.532), p=0.001 *	261.363 (78.887-443.838), p=0.007 *	25.196 (-45.25-95.643), p=0.487	1.634 (-1.242-4.51), p=0.271	-1.375 (-3.938-1.187), p=0.298	-13.175 (-34.03-7.681), p=0.222	1.112 (-2.793-5.017), p=0.579	1.048 (-4.701-6.796), p=0.723	-118.53 (-317.993-80.933), p=0.25	-228.597 (-352.998--104.196), p=0.001 *
OSI NWS	-0.483 (-50.243-49.278), p=0.985	-13.266 (-63.143-36.612), p=0.605	-0.429 (-19.685-18.827), p=0.965	-0.198 (-0.984-0.588), p=0.624	-0.171 (-0.872-0.529), p=0.634	-0.242 (-5.942-5.459), p=0.934	0.099 (-0.968-1.167), p=0.856	-0.609 (-2.18-0.962), p=0.451	18.549 (-35.972-73.07), p=0.508	-12.536 (-46.539-21.468), p=0.473

Parameter	Hemorrhagic stroke	Ischemic stroke	Gender: Male	Age [yrs]	ACE III	ADL	FIM	Berg	NWS flow	SWS flow
OSI SWS	26.739 (-29.313-82.792), p=0.354	37.632 (-18.553-93.816), p=0.195	27.649 (5.958-49.339), p=0.016 *	0.275 (-0.61-1.161), p=0.545	0.061 (-0.728-0.85), p=0.879	-1.294 (-7.715-5.128), p=0.695	-0.939 (-2.141-0.264), p=0.132	1.814 (0.044-3.584), p=0.05	47.964 (-13.45-109.379), p=0.132	-45.342 (-83.645--7.039), p=0.025 *
Px NWS	0.014 (-0.018-0.046), p=0.395	0.024 (-0.008-0.056), p=0.143	0.004 (-0.009-0.016), p=0.573	0 (0-0.001), p=0.217	0 (-0.001-0), p=0.155	-0.002 (-0.006-0.002), p=0.291	0 (-0.001-0), p=0.29	0.001 (0-0.002), p=0.022 *	-0.003 (-0.037-0.032), p=0.877	0.019 (-0.003-0.041), p=0.093
Px SWS	0.043 (0.006-0.08), p=0.027 *	0.041 (0.004-0.078), p=0.036 *	-0.002 (-0.016-0.012), p=0.781	0 (-0.001-0.001), p=0.815	0 (-0.001-0), p=0.151	0 (-0.004-0.005), p=0.85	-0.001 (-0.002-0), p=0.081	0.001 (0-0.002), p=0.116	0.032 (-0.008-0.073), p=0.125	0.013 (-0.012-0.038), p=0.32
SOD NWS	5.102 (-0.937-11.142), p=0.104	3.262 (-2.791-9.316), p=0.296	-1.435 (-3.772-0.903), p=0.235	0.045 (-0.05-0.141), p=0.359	0.018 (-0.067-0.103), p=0.677	0.217 (-0.475-0.909), p=0.542	-0.083 (-0.212-0.047), p=0.216	0.099 (-0.091-0.29), p=0.312	-1.167 (-7.785-5.45), p=0.731	-1.165 (-5.292-2.962), p=0.583
SOD SWS	-5.335 (-15.317-4.646), p=0.3	-3.996 (-14.001-6.009), p=0.437	3.372 (-0.491-7.234), p=0.093	0.112 (-0.046-0.27), p=0.17	0.022 (-0.119-0.162), p=0.765	-0.504 (-1.648-0.639), p=0.392	0.088 (-0.126-0.302), p=0.423	-0.036 (-0.351-0.279), p=0.824	-5.642 (-16.578-5.294), p=0.317	0.057 (-6.763-6.878), p=0.987
TAC NWS	0.415 (-1.055-1.884), p=0.583	0.691 (-0.783-2.164), p=0.363	0.087 (-0.482-0.655), p=0.766	0.011 (-0.012-0.034), p=0.359	-0.004 (-0.025-0.017), p=0.704	-0.076 (-0.245-0.092), p=0.38	-0.014 (-0.045-0.018), p=0.404	0.046 (0-0.092), p=0.058	-0.8 (-2.411-0.81), p=0.335	1.064 (0.059-2.068), p=0.043 *
TAC SWS	-1.034 (-2.766-0.698), p=0.248	-0.577 (-2.313-1.159), p=0.518	-0.761 (-1.431--0.091), p=0.031 *	-0.002 (-0.03-0.025), p=0.86	0.005 (-0.019-0.03), p=0.672	-0.162 (-0.36-0.037), p=0.116	0.043 (0.005-0.08), p=0.029 *	-0.074 (-0.129--0.019), p=0.011 *	0.282 (-1.615-2.18), p=0.772	0.917 (-0.266-2.101), p=0.135
TOS NWS	28.955 (-41.757-99.667), p=0.426	14.677 (-56.201-85.555), p=0.687	-3.202 (-30.565-24.161), p=0.82	0.168 (-0.949-1.285), p=0.769	-0.203 (-1.198-0.793), p=0.691	-2.668 (-10.769-5.433), p=0.522	-0.818 (-2.335-0.699), p=0.296	1.275 (-0.958-3.508), p=0.268	-25.843 (-103.319-51.633), p=0.516	27.813 (-20.508-76.133), p=0.265
TOS SWS	5.794 (-162.608-174.197), p=0.947	56.645 (-112.153-225.443), p=0.514	35.569 (-29.598-100.735), p=0.29	1.312 (-1.348-3.973), p=0.338	0.497 (-1.874-2.867), p=0.683	-15.285 (-34.578-4.008), p=0.127	-0.286 (-3.899-3.326), p=0.877	0.46 (-4.858-5.777), p=0.866	122.354 (-62.158-306.866), p=0.2	-113.307 (-228.383-1.77), p=0.059
UA NWS	6.179 (-12.896-25.253), p=0.528	4.604 (-14.515-23.724), p=0.639	1.565 (-5.817-8.946), p=0.68	0.114 (-0.187-0.416), p=0.46	-0.125 (-0.394-0.143), p=0.364	-0.189 (-2.374-1.996), p=0.866	0.163 (-0.246-0.572), p=0.439	-0.294 (-0.897-0.308), p=0.343	2.571 (-18.329-23.47), p=0.811	-7.658 (-20.692-5.377), p=0.255
UA SWS	-9.506 (-42.088-23.076), p=0.57	-0.796 (-33.455-31.862), p=0.962	8.128 (-4.48-20.736), p=0.212	-0.132 (-0.647-0.383), p=0.618	-0.054 (-0.512-0.405), p=0.819	-3.013 (-6.745-0.72), p=0.12	0.064 (-0.635-0.763), p=0.858	0.299 (-0.73-1.328), p=0.572	10.685 (-25.014-46.384), p=0.56	5.158 (-17.107-27.423), p=0.652

* statistically significant (p<0,05).
